# The Alteration of Subtelomeric DNA Methylation in Aging-Related Diseases

**DOI:** 10.3389/fgene.2018.00697

**Published:** 2019-01-09

**Authors:** Haochang Hu, Bin Li, Shiwei Duan

**Affiliations:** Medical Genetics Center, School of Medicine, Ningbo University, Ningbo, China

**Keywords:** epigenetic modification, subtelomeric DNA methylation, telomere length, telomerase, age-related disease

## Abstract

The telomere is located at the end of the chromosome and consists of a non-coding, repetitive DNA sequence. As the cell divides, the length of telomere gradually decreases. A very short telomere can terminate mitosis, and thus telomere length becomes a hallmark of cellular aging. The 500 kb region of each autosomal arm terminal is the so-called subtelomeric region. Both telomere and subtelomere have high-density DNA repeats. Telomeres do not contain genes or CpG sequences, while subtelomeres contain small amounts of genes and high-density CpG sequences, and DNA methylation often occurs in subtelomeres. Previous studies have shown that aberrant methylation of subtelomeric DNA exists in many diseases, and it has a certain effect on the regulation of telomere length. In this review, we focus on the correlation between subtelomeric DNA methylation and aging-related diseases. We also summarize the relationship between subtelomeric methylation and telomere length in different diseases.

## Introduction

In the 1930s, people began to understand telomere, a special structure on the chromosome. With continued cell division, telomeres gradually shorten, and a very short telomere terminates mitosis (Harley et al., 1990). When a cell stops replicating, it enters a period called “cell decay,” and thus telomere length becomes a hallmark of cellular aging. In addition, the detection of telomere length can also help us assess the risk of aging disease (Herrmann et al., 2018).

DNA methylation is prevalent in the subtelomeric DNA region of mammals. This epigenetic phenomenon may play an important role in transcriptional regulation and chromosomal structural remodeling by regulating DNA binding factors.

Recent studies have shown that subtelomeric DNA methylation changes in many diseases, which are closely related to telomere length control mechanisms. In this review we focus on the correlation of subtelomeric DNA methylation with aging-related diseases. We also check the relationship between subtelomeric methylation and telomere length in different diseases.

## Telomeres, Telomerase Activity and Telomere Length

**FIGURE 1:**
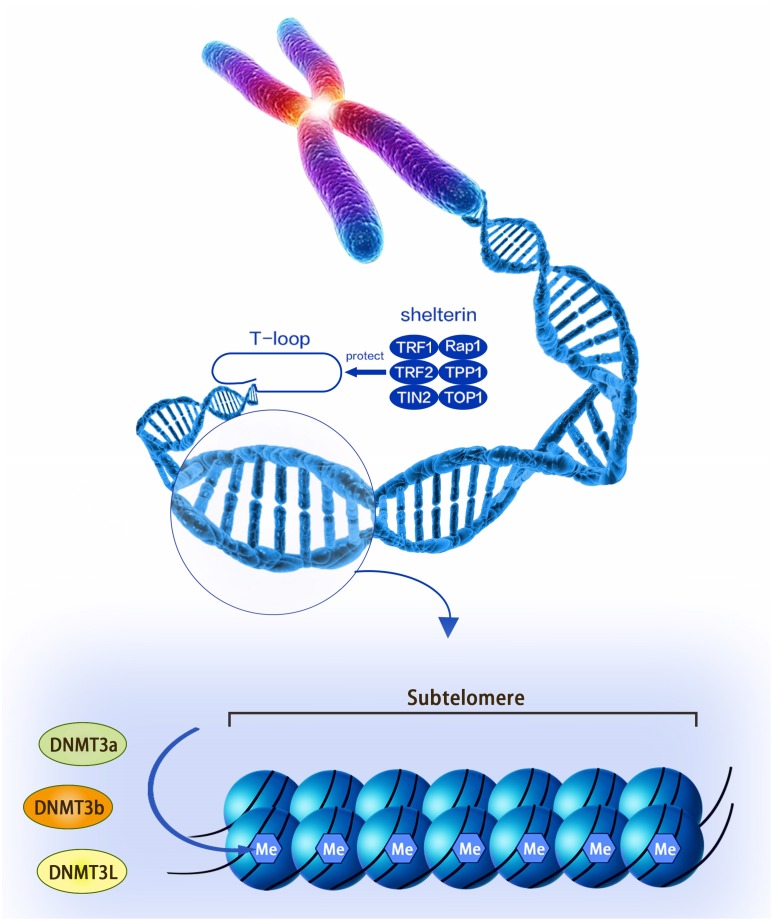
A structural diagram of telomeres and subtelomeres. The methylation level of the subtelomeric DNA can be regulated by the DNMT3L, DNMT3a, and DNMT3b enzymes.

Telomere was first discovered by Hermann in 1938, and it is a nuclear protein structure at the end of the chromosome (Herrmann et al., 2018). Telomeres are protective caps at the ends of chromosomes in cells, and they are composed of non-coding, repetitive DNA sequences and telomere-binding proteins. Telomere DNA sequences are both highly conserved and species-specific, and each organism has its own specific sequence and average length. The human and mammalian telomere sequence is -TTAGGG- (Steinert et al., 2004). The telomere-binding protein is a complex of six proteins named shelterin that consists of TRF1, TRF2, TIN2, Rap1, TPP1, and POT1. The complex not only prevents the activation of the DNA damage reaction at the end of the chromosome, but also regulates the telomerase activity at the end of the chromosome, thereby maintaining the stability of the T-loop structure. The T-loop structure is the single-stranded fold protruding at the 3′ end and invading the telomere double helix structure. This structure facilitates the formation of a cap structure at the end of the chromosome by the telomere (Figure 1). Telomere dysfunction leads to the fusion of the ends of the chromosome, leading to chromosomal instability (Oh et al., 2011).

Telomerase is a special ribonucleoprotein protease complex with reverse transcriptase activity. It uses a primer-specific recognition site to synthesize telomeric DNA at the end of the chromosome and extends its telomere with its own RNA as a template. Telomere repeat amplification protocol (TRAP) assay is a commonly used routine assay with high sensitivity for telomerase activity (Kim et al., 1994). Due to PCR inhibition by biomolecules such as bile salt, hemoglobin, heparin, and lactoferrin, TRAP assay is prone to false-negative results (Kim and Wu, 1997). Although other telomerase assays based on various sensors or methods are developed, these assays are less sensitive than TRAP assay (Yaku et al., 2013). Fortunately, the integration of TRAP internal controls helps to measure telomerase activity levels, and the false negative results of telomerase-positive samples are extremely low (Kim and Wu, 1997). Telomerase assay with high sensitivity and low false negative results should be explored in the future.

The length of telomeres can be thought as the individual’s biological clock. There are different strategies for determining telomere length, including terminal restriction fragment (TRF), Southern blotting (SB) analysis, quantitative polymerase chain reaction (PCR), and flow-fluorescence *in situ* hybridization (FISH) (Tarik et al., 2018). TRF analysis is the oldest approach for determining telomere length. Without enzyme-cutting sites in the telomeric and subtelomeric regions, TRF is always considered as “gold standard” method (Tarik et al., 2018). For a large number of samples, PCR-based assays (including quantitative PCR, monochromatic multiplex quantitative PCR (MMqPCR) and absolute telomere length (aTL) quantification were applied to telomere length assessment. Unlike the assays described above, the substrate for quantitative-FISH (Q-FISH) is cells rather than DNA (Montpetit et al., 2014).

There are two mechanisms for telomere shortening, one of which is due to the inhibition of telomerase, resulting in incomplete DNA replication at the end of the chromosome, i.e., “end replication problem” (Guan et al., 2013). Cancer cells can also get rid of the limitations of aging by raising telomerase to maintain telomere levels (Shay and Bacchetti, 1997). In some cases, telomere length can be maintained by another mechanism, namely alternative lengthening of telomeres (ALT), which is based on homologous recombination-mediated DNA replication in the telomere sequence (Lee et al., 2009).

The somatic telomeres in the peripheral blood of the elderly are shorter than those in the young (Ye et al., 2018). In addition, telomere shortening is also accelerated by various pathophysiological conditions, including physical stress and disease state (Maeda et al., 2012). For example, according to the results of Kheirollahi, higher grade meningiomas and astrocytoma tumors show more heterogeneity in telomere length (Kheirollahi et al., 2011). Besides, telomere length is shortened and triggers chromosome instability in the early development of brain tumor (Nasser and Mehdipour, 2018).

## Subtelomeres and Subtelomeric DNA Methylation

The vicinity of the telomeres is the so-called subtelomeric region, which is defined as 500 kb of each autosomal arm terminal (Macina et al., 1994). In 2004, Susann et al. discovered a subtelomeric DNA fragment called X-region in Hela cells. The researchers sequenced human subtelomeric sequences and found 941 transcripts, including 214 single-copy genes (Riethman et al., 2004). Both telomere and subtelomeric sequences have high-density DNA repeats, but telomeres do not contain genes or CpG sequences, while subtelomeres contain small amounts of genes as well as high-density CpG sequences (Blasco, 2007). The telomeric DNA and their related proteins play an important role in genome stability and chromosome replication. Similarly, subtelomeric DNA fragments can partially regulate key biological activities, including cell cycle regulation, cell senescence and immortalization, movement and localization of chromosomes within the nucleus, and transcription of subtelomeric genes (Riethman et al., 2005). For example, subtelomeric abnormalities may have significant effects on idiopathic mental retardation and several mental retardation syndromes (Hila et al., 2009). Previous studies have shown that 6p subtelomeric deletions have occurred in growth stunting or mental retardation (DeScipio, 2007; Nakane et al., 2013). In addition, Wu et al. (2010) found that 16 patients with growth stunting or mental retardation had several deletions at 14 different subtelomeric regions (1p, 2p, 4p, 6p, 7p, 7q, 8p, 9p, 10p, 11q, 14q, 15q, 16p, and 22q) and repeats at seven subtelomeric regions (3q, 4p, 6q, 7p, 8p, 12p and 22q). What’s more, subtelomeric rearrangements are also the major cause of idiopathic mental retardation, growth stunting or retardation, and malignant blood system diseases (Ravnan et al., 2006; Tos et al., 2013; El-Hajj Ghaoui et al., 2017).

Recent studies have found that epigenetic modification acted as a bridge between genetic and environmental factors and played an important role in the occurrence and development of diseases (Lovinsky-Desir and Miller, 2012; Hu et al., 2017). The regulatory mechanisms of epigenetics mainly include DNA methylation, histone modification, non-coding RNA action, and etc. DNA methylation, as one of the important mechanisms of epigenetics, has many important physiological significances *in vivo*. Normal methylation is essential for maintaining cell growth and metabolism, while abnormal DNA methylation causes disease (such as tumors) (Vera et al., 2008). The apparent modification of subtelomeres has long been of concern to scientists, and in 1990, *de novo* methylation was first found at a subtelomeric repeat with the minimal (4 kb) in somatic cells (de Lange et al., 1990). The subtelomeric region is rich in CpG islands, so DNA methylation often occurs in this region. A systematic search revealed a rare DNA methyltransferase 3L (DNMT3L) variant that may affect the structure or function of the corresponding DNMT protein associated with subtelomeric hypomethylation (El-Maarri et al., 2009). During early development, the subtelomeric region was methylated by DNMT3b (Walton et al., 2011; Simo-Riudalbas et al., 2015). At a critical stage in human embryonic development, subtelomeric DNA methylation may be necessary for maintaining normal telomere length (Yehezkel et al., 2013).

## Subtelomeric DNA Methylation and Telomere Length

Subtelomeric methylation is thought to be closely related to telomere length. Focusing on nucleotide sequences, the subtelomere has a high density of CpG sequences that are susceptible to methylation of DNMT (including DNMT1, DNMT3a, and DNMT3b) (Figure 1; Brock et al., 1999; Steinert et al., 2004; Blasco, 2007). Past reports indicated that the subtelomeric region in mice was largely methylated and this epigenetic modification was reduced in DNMT-deficient embryonic stem (ES) cells. Interestingly, this genetically deficient cell significantly prolonged telomeres compared to wild-type controls (Gonzalo et al., 2006). The following experiments demonstrated that this phenomenon was caused by increased homologous recombination in the telomere sequence, whereas the introduction of DNMTs 3a and 3b into DNMTs-deficient cells restored telomere methylation and reduced homologous recombination of telomere sequences (Gonzalo et al., 2006). Therefore, DNA methylation in the subtelomeric repeat region has the effect of inhibiting telomere homologous recombination and may affect the ALT mechanism (Gonzalo et al., 2006). Similarly, as telomeres shorten, subtelomeric chromatin also undergoes a conformational transition to a more commonly stained state, accompanied by decreased methylation and increased acetylation in telomerase knockout mouse cells. This process is thought to promote the recruitment of telomerase (Benetti et al., 2007; Blasco, 2007). Another mouse model found that there was always more dense methylation in the telomere-positive cells compared to normal cells. At the same time, telomerase-positive cells can promote telomerase maintenance of telomere length by reducing the level of telomere-containing repeat RNA (TERRA), the transcription of which originates from the subtelomere (Ng et al., 2009).

In recent years, scientists have begun to explore the association between methylation of specific subtelomeric CpG sites, telomere length and gene expression. Buxton’s study found that methylation at many gene promoter regions distributed in the subtelomeric region was positively correlated with telomere length in human leukocyte DNA. And shorter telomeres are significantly associated with decreased methylation levels at most of these sites. Therefore, the authors speculated that with the shortening of telomeres, the methylation levels of the subtelomeric regions of many gene promoters may change, which in turn may lead to changes in gene expression and increase the risk of age-related diseases (Buxton et al., 2014). In addition, Choudhury et al. (2016) used optogenetics to increase DNA methylation at specific subtelomeric CpG sites, resulting in a gradual increase in telomere length during replication of three generations of HeLa cells.

## Subtelomeric DNA Methylation and Aging-Related Diseases

In epidemiological studies, individuals with shorter telomeres have a higher mortality rate, almost twice as many as those with longer telomeres (Goglin et al., 2016). A large number of previous studies have shown that short telomere and telomere dysfunction were associated with many diseases. For example, a shorter telomere length may indicate a higher risk of aging-related disease in patients (Herrmann et al., 2018).

It has been reported that abnormal epigenetic changes in subtelomeric CpG islands affected several biological pathways or induced cell reprogramming due to induction of methylation or hydroxymethylation, however, the specific mechanisms still need to be explored (Wang et al., 2013). Previous studies have shown that epigenetic modification of subtelomere had a certain effect on the regulation of telomere length (Table 1). And the mouse-based model described above also suggested a conserved link between telomere length and the epigenetic state of the subtelomere. Therefore, we hypothesized that subtelomeric DNA methylation may cause aging disease by affecting telomere length.

**Table 1:** The correlation between subtelomeric methylation and telomere length in aging-related diseases.

Disease	The location of subtelomeric region	The methylation status of subtelomeric region	The correlation between subtelomeric methylation and telomere length	Reference
Glioblastomas	Chr. 4q (D4Z4)	Demethylation	NA	Cadieux et al., 2006
Glioblastomas	Chr. 10q	Hypomethylation	NA	Cadieux et al., 2006
Lung cancer	Chr. 8p	Hypomethylation	NA	Rauch et al., 2008
Hepatocellular carcinoma	Chr. 7q	Hypomethylation	Irrelevant	Oh et al., 2011
Hepatocellular carcinoma	Chr. 18p	Hypermethylation	Negative	Oh et al., 2011
Hepatocellular carcinoma	Chr. 21q	Hypermethylation	Positive	Oh et al., 2011
Astrocytoma	Chr. 2p	Hypermethylation	NA	Lyke et al., 2012
Nasopharyngeal carcinoma	Chr. 4q35 (D4Z4)	Demethylation	Positive	Zhang et al., 2014
Glioblastomas	Chr. 8q	Hypermethylation	NA	Choudhury et al., 2015
Glioblastomas	Chr. 21q	Hypermethylation	NA	Choudhury et al., 2015
Glioblastomas	Chr. XpYp	Hypermethylation	NA	Choudhury et al., 2015
Gallbladder carcinoma	Chr. 4q35 (D4Z4)	Hypermethylation	Irrelevant	Poojary et al., 2016
Alzheimer’s disease	NA	Hypermethylation	NA	Guan et al., 2013
Parkinson’s disease	NA	Hypermethylation	Negative	Maeda et al., 2009
Hypertension	NA	Hypermethylation	Negative	Maeda et al., 2011b


*NA: not available.*

As the elderly population increases, the incidence of aging-related diseases is increasing, such as cancer, cardiovascular disease (CVD), type 2 diabetes (T2DM), neurodegenerative diseases, osteoporosis and premature aging. The consumption of telomeres is related to the aging process and to the condition of senile diseases. At the same time, the process of aging also affects the methylation status of the subtelomere.

## Cancer

The risk of developing cancer increases along with aging, and thus cancer is defined as one of the common age-related diseases (Garrido and Djouder, 2017). One of the causes of cancer is the widespread distribution of DNA methylation in genomic chromatin. These changes often affect the structure of chromatin, which may cause abnormal expression of some oncogenes, leading to carcinogenesis (Klutstein et al., 2016; Li et al., 2018). Epigenetic modification of subtelomere can also lead to the similar consequences.

A large number of studies have validated the relationship between subtelomeric DNA methylation and the risk and prognosis of cancer. Poojary et al. (2016) found that in early gallbladder cancer, a chromosome 4q35 subtelomeric sequence (D4Z4) was hypermethylated and the telomere length was significantly shortened. However, no correlation was found between subtelomeric methylation and telomere length. In gliomas, their subtelomeric DNA methylation levels were consistently higher than in the control group, especially in the subtelomeric regions of chromosomes 8q, 21q, and XpYp, regardless of patient age and tumor grade (Choudhury et al., 2015). Zhang et al. (2014) evaluated the role of demethylation drug (5-AZA) in nasopharyngeal carcinoma cells (CNE, CNE1, CNE2, and 5-8F). They found that 5-AZA induced significant demethylation and inhibited the expression of the human telomerase catalytic subunit (hTERT), which reduced telomerase activity and significantly shortened telomere length. In nasopharyngeal carcinoma, demethylation of the chromosome 4q35 subtelomeric region (D4Z4) may result in decreased expression of hTERT and shortening of telomeres. However, in the demethylation experiment of malignant glioma cell lines, telomere length did not change with changes in subtelomeric DNA methylation (Choudhury et al., 2015). Yan et al. (2012) studied the genome-wide activity of decitabine in acute myeloid leukemia and found that decitabine preferred to demethylate the CpG islands in subtelomeres. And decitabine had different activities in different chromosomal regions, which provided a theoretical basis for demethylating drugs to treat diseases (Yan et al., 2012). In addition, Myung et al. found no significant association between telomere length and subtelomeric DNA methylation in human tumor cells by MSP and pyrosequencing (Lee et al., 2009).

High-throughput whole-genome sequencing has shown significantly hypomethylated subtelomeric regions on chromosomes 4q and 10q for malignant glioma (Cadieux et al., 2006) and chromosome 8p for lung cancer (Rauch et al., 2008). Liver cancer showed a higher methylation rate in the 18p and 21q subtelomeric regions compared to the control group. Interestingly, in patients with liver cancer, subtelomeric methylation at chromosome 18p was inversely correlated with telomere length; while subtelomeric methylation at chromosome 21q was positively correlated to telomere length (Oh et al., 2011). During the development of liver cancer, the telomere length is prolonged, accompanied by hypomethylated subtelomeric region at chromosome 7q and hypermethylated subtelomeric region at chromsome 21q (Oh et al., 2011). The methylation changes of different subtelomeres are different, which indicates that the relationship between human subtelomeric methylation and telomere length is complicated.

Transcription from subtelomeres produces TERRA, a natural inhibitor of telomerase activity (TA). In astrocytoma, DNA methylation of subtelomeric CpG island is associated with low expression of TERRA on the chromosome 2p (Sampl et al., 2012). This suggests that the expression of TERRA may be specific to the regulation of the subtelomeric methylation at one chromosome.

In prostate cancer, hypermethylation of the subtelomeric region D4Z4 was found to be associated with the worse prognosis of the patients (Han et al., 2017). We hypothesize that subtelomeric DNA methylation may be a potential biomarker for tumor prognosis, and more experiments should be performed to validate this conjecture.

## Neurodegenerative Diseases

Alzheimer’s disease (AD) and Parkinson’s disease (PD) are the two most common chronic neurodegenerative diseases associated with aging. AD is characterized by a gradual decline in cognitive and memory functions. It is estimated that by 2050, the number of people affected by AD worldwide will reach 150 million^1^. Guan et al. (2013) found that in patients with AD, the subtelomere methylation of patients with short telomeres (<4.4 kb) was higher than that of patients with long telomeres. At the same time, they also found that leukocytes with short-telomeric DNA hypomethylation tend to be cleared more quickly from the peripheral blood of AD patients, resulting in normal telomere length in AD patients (Guan et al., 2012b). In addition, they also observed the role of antioxidant vitamin E in patients with AD, and found that vitamin E could reduce the level of oxidative stress in AD patients, however, vitamin E had no significant effect on the telomere length of AD patients (Guan et al., 2012a).

Parkinson’s disease (PD) is a neurodegenerative disease described as “shaking paralysis.” Clinical features of PD include motor symptoms such as tremor, muscle stiffness, dyskinesia, postural instability, cognitive symptoms, and circadian rhythm disorders. It is estimated that among the 10 countries with the largest number of people in the world, the incidence of idiopathic Parkinson’s disease in 2030 is expected to reach 8.7 to 9.3 million (Allyson Jones et al., 2012). Maeda et al. (2009) found a significant correlation between telomere shortening and subtelomeric methylation in PD. As PD-related neurodegeneration occurs, the state of telomeres and subtelomeres changes. Compared with the healthy control group, Japanese women with PD had a decrease in leukocytes with long telomeres and a proportional increase of hypomethylated subtelomeres in leukocytes with short telomeres (Maeda et al., 2012). PD also causes an increase in telomere fragility, which accelerates telomere shortening in leukocytes. Future experiments should be performed in more neurodegenerative diseases.

## Cardiovascular and Cerebrovascular Diseases

The incidence and mortality of CVD is extremely high, and the incidence will increase further in both developing and developed countries in the future. The pathophysiological features of CVD are endothelial dysfunction, vascular inflammation, atherosclerosis, fibrosis, and thrombosis. Studies have shown that telomere shortening was associated with the risk of coronary heart disease, myocardial infarction, heart failure, and stroke (Willeit et al., 2010). In hypertensive patients, the degree of subtelomeric methylation of long telomeres was often inversely correlated with telomere consumption (Maeda et al., 2011b). Researchers found that cardiac function was related to both telomere length and subtelomeric DNA methylation (Maeda et al., 2018). Maeda et al. (2013) also found that subtelomeres were hypomethylated and telomerase activity was decreased in senescent smooth muscle cells.

Cerebrovascular disease refers to various diseases of the blood vessels in brain, including cerebral atherosclerosis, thrombosis, stenosis, occlusion, cerebral arteritis, cerebral artery injury, cerebral aneurysm, intracranial vascular malformation, and cerebral arteriovenous fistula. Common features are caused by ischemia or hemorrhagic accidents in brain, resulting in disability or death of the patient. Maeda et al. (2010) studied the association of telomere length and subtelomeric DNA methylation with clinical laboratory data in patients with cerebral infarction and metabolic disorders. There was a significant correlation of fasting blood glucose, HbA1c, serum creatinine and urea nitrogen levels with telomere length and subtelomeric DNA hypermethylation. The physiological capacity of patients with cerebrovascular disease was positively correlated with telomere length, and the recovery of physiological ability was related to subtelomeric DNA hypermethylation (Maeda et al., 2011a).

Cardiovascular and cerebrovascular diseases are common diseases that seriously threaten the health of humans, especially middle-aged and elderly people. Previous studies have shown that cardiovascular and cerebrovascular diseases were significantly associated with subtelomeric DNA methylation and telomere length (Maeda et al., 2011b, 2018). In the future, subtelomeric DNA methylation should be investigated in other cardiovascular and cerebrovascular diseases (such as coronary heart disease, myocardial infarction, stroke, and etc.).

## Conclusion

A growing body of experimental evidence indicates a significant change in the subtelomeric DNA methylation in age-related diseases. For example, subtelomeric DNA hypomethylation was found in lung cancer, malignant glioma, PD and other diseases, and subtelomeric DNA hypermethylation was found in diseases such as gallbladder cancer and AD. However, the methylation status of telomeres should be repeatedly demonstrated in large samples to arrive at credible conclusions.

The degree of subtelomeric methylation on different chromosomes or different arms in the same chromosome may not be the same. With the development of advanced technologies, FISH, an accurate molecular cytogenetic tool, uses fluorescently labeled DNA probes to identify microchromosomal changes that contain chromosomal rearrangements in the subtelomeric region (Meloni et al., 1993; Peterson et al., 2018). It reveals the diversity behavior of the p- and q-chromosomal arm signals. Recent FISH-based studies have shown that the reduced 5′RARA signal and the single PML-RARA fusion are located in the subterminal q-arm and p-arm regions of the rearranged chromosome 17, respectively (El-Hajj Ghaoui et al., 2017). Whether in a neoplastic disease or a non-cancerous disease, disease progression depends on the quantification and quality of the signal, and these signals differ between the p- and q-arms of each chromosome. Therefore, we should explore the quantity and quality of signals in different parts of each chromosome.

Furthermore, DNA methylation changes in the same subtelomere may not be similar in different age-related diseases. Interestingly, subtelomeric DNA hypomethylation at one site was not accompanied by a decrease in the global subtelomeric DNA methylation (Gonzalo et al., 2006). These results indicate DNA methylation changes in the subtelomeric loci may be specific to different disease. Unfortunately, up to now, there have been few studies on DNA methylation of specific subtelomeric regions, and this may be one of the next directions of subtelomeric researches. And subtelomeric DNA methylation in specific sites can be used as an indicator of age-related disease progression. Then, due to the complexity of age-related diseases, heterogeneity cannot be ignored, so multi-Omics should be used to find out the relevant mechanisms.

As telomere research continues to heat up, according to the present study, changes in telomere length associated with subtelomeric DNA methylation are considered to be important processes in the pathogenesis of aging-related diseases. In the previous concept, the association between subtelomeric DNA methylation and telomere length was not identical in similar diseases. In this review, the relationship between subtelomeric DNA methylation and telomere length is discussed. Some age-related diseases were positively correlated with subtelomeric DNA methylation and telomere length, and negatively correlated with other age-related diseases, and the other parts were not significantly correlated. This makes it harder for us to study the correlation between telomere length and subtelomeric DNA methylation.

In addition, subtelomeric methylation and telomere length interact in some cases and have created a potential mechanism for several interactions. First, DNA methylation in the subtelomeric repeat region has the effect on inhibiting telomere homologous recombination and may affect ALT mechanisms to alter telomere length (Gonzalo et al., 2006). Second, telomerase-positive cells may alter the methylation level of the subtelomeric region by lowering TERRA levels, which may inhibit telomerase to maintain telomere length (Ng et al., 2009). Third, as telomere shortens, subtelomeric chromatin is accompanied by decreased methylation, and telomerase knockout mice have increased acetylation, which is thought to be beneficial for telomerase recruitment (Benetti et al., 2007; Blasco, 2007). However, due to its complex etiology, the exact mechanism of age-related diseases is difficult to fully explain. Multi-Omics is used to increase the likelihood of a potential mechanism for determining the relationship between subtelomeric DNA methylation and telomere length through integration interactions and corresponding correlations. In addition, research on human transformation through *in vivo* and/or *in vitro* should be fully revealed in the future.

In addition, a large number of diseases are closely related to genomic risk factors, and attention to genomic levels provides a large number of clues for pathogenesis and drug targets. Researchers reduced subtelomeric DNA methylation by demethylating drugs, and hoped to negatively regulate the telomere length of tumor cells by DNA methylation, thereby reducing the stability of tumor cell genome and helping clinical diagnosis and treatment of tumors. Apart from demethylating drugs, regulation of telomere length by re-expression of telomerase or inhibition of telomerase may be a potential treatment for disease.

Finally, our review suggests that subtelomeric DNA methylation is highly associated with aging diseases. However, its role in the development of aging diseases is not totally unmasked, and therefore, the role of subtelomeric DNA methylation in the development of aging diseases will be further evaluated in the future.

## Author Contributions

SD and HH contributed to the conception, design, writing and final approval of the submitted version. HH and BL contributed to the revision of manuscript.

## Conflict of Interest Statement

The authors declare that the research was conducted in the absence of any commercial or financial relationships that could be construed as a potential conflict of interest.

## Footnotes

1 https://www.alz.co.uk/research/world-report-2018
